# Verotoxin Receptor-Based Pathology and Therapies

**DOI:** 10.3389/fcimb.2020.00123

**Published:** 2020-03-31

**Authors:** Clifford Lingwood

**Affiliations:** Molecular Medicine, Research Institute, Hospital for Sick Children, Toronto, ON, Canada

**Keywords:** lipid raft, retrograde transport, endothelial cell, astrocyte, hemolytic uremic syndrome

## Abstract

Verotoxin, VT (aka Shiga toxin,Stx) is produced by enterohemorrhagic *E. coli* (EHEC) and is the key pathogenic factor in EHEC-induced hemolytic uremic syndrome (eHUS-hemolytic anemia/thrombocytopenia/glomerular infarct) which can follow gastrointestinal EHEC infection, particularly in children. This AB5 subunit toxin family bind target cell globotriaosyl ceramide (Gb_3_), a glycosphingolipid (GSL) (aka CD77, pk blood group antigen) of the globoseries of neutral GSLs, initiating lipid raft-dependent plasma membrane Gb_3_ clustering, membrane curvature, invagination, scission, endosomal trafficking, and retrograde traffic via the TGN to the Golgi, and ER. In the ER, A/B subunits separate and the A subunit hijacks the ER reverse translocon (dislocon-used to eliminate misfolded proteins-ER associated degradation-ERAD) for cytosolic access. This property has been used to devise toxoid-based therapy to temporarily block ERAD and rescue the mutant phenotype of several genetic protein misfolding diseases. The A subunit avoids cytosolic proteosomal degradation, to block protein synthesis via its RNA glycanase activity. In humans, Gb_3_ is primarily expressed in the kidney, particularly in the glomerular endothelial cells. Here, Gb_3_ is in lipid rafts (more ordered membrane domains which accumulate GSLs/cholesterol) whereas renal tubular Gb_3_ is in the non-raft membrane fraction, explaining the basic pathology of eHUS (glomerular endothelial infarct). Females are more susceptible and this correlates with higher renal Gb_3_ expression. HUS can be associated with encephalopathy, more commonly following verotoxin 2 exposure. Gb_3_ is expressed in the microvasculature of the brain. All members of the VT family bind Gb_3_, but with varying affinity. VT2e (pig edema toxin) binds Gb_4_ preferentially. Verotoxin-specific therapeutics based on chemical analogs of Gb_3_, though effective *in vitro*, have failed *in vivo*. While some analogs are effective in animal models, there are no good rodent models of eHUS since Gb_3_ is not expressed in rodent glomeruli. However, the mouse mimics the neurological symptoms more closely and provides an excellent tool to assess therapeutics. In addition to direct cytotoxicity, other factors including VT–induced cytokine release and aberrant complement cascade, are now appreciated as important in eHUS. Based on atypical HUS therapy, treatment of eHUS patients with anticomplement antibodies has proven effective in some cases. A recent switch using stem cells to try to reverse, rather than prevent VT induced pathology may prove a more effective methodology.

## Introduction

Originally termed Verotoxin (VT), because of its discovery as a novel enterohemorrhagic *E coli* (O157:H7) derived toxin effective against the vero African green monkey kidney cell line (Konowalchuk et al., 1977), but currently more frequently termed Shiga toxin or Shiga-like toxin due to its single amino acid difference with Shiga toxin (Stx) from *Shigella dysenteriae* (DeGrandis et al., 1987).

EHEC produced Verotoxin 1 was shown, in Karmali's landmark studies (Karmali et al., 1985), to be responsible for the hemolytic uremic syndrome, a renal glomerular pathology with a triad of symptoms: thrombocytopenia, hemolytic anemia, and glomerular endothelial infarct, with no previously defined cause. Unfortunately, antibiotic treatment increases rather than reduces pathology (Zhang et al., 2000). Females are more susceptible and this correlates with increased renal Gb_3_ (Fujii et al., 2016). With a fatality rate of ~10% and highest incidence in the pediatric and elderly population, it is of concern that since the infectious cause was defined 35 years ago, no specific therapeutic approach has been achieved.

## EHEC Toxins

The Shiga (vero) toxins are a family of AB5 bacterial subunit toxins, primarily comprising VT1 and VT2 (Nakao and Takeda, 2000) [though many other minor variants of VT2 are known (Zhang et al., 2008)]. VT2 is 60% identical but significantly less toxic than VT1 in cell culture (Fuller et al., 2011). VT2 binds Gb_3_ with lower affinity (Nakajima et al., 2001) but VT2 causes increased toxicity in mice (Conrady et al., 2010; Fuller et al., 2011) and other animal models (Mizuguchi et al., 2001; Takahashi et al., 2008) with increased association with disease (Kawano et al., 2008), particularly neurological sequelae following EHEC infection (Trachtman et al., 2012; Kramer et al., 2015). The different toxicity potency is due to B subunit differences (Head et al., 1991).

## VT Receptor

The receptor binding pentameric B subunit of Shiga toxin binds the neutral glycosphingolipid (GSL), globotriaosyl ceramide (Gb_3_, aka CD77 and the p^k^ blood group antigen) (Lingwood et al., 1987), which is highly expressed in the human kidney (Boyd and Lingwood, 1989; Lingwood, 1994). GSLs are sugar-ceramide conjugates, for the most part, based on the ceramide monohexoside, glucosyl ceramide. Single sugar units are added in α or β anomeric linkage to form linear or branch chain GSLs in the Golgi membrane, which are then transported to the plasma membrane by vesicular traffic. Over 400 carbohydrate structures have been defined (Stults et al., 1989). The structure of Gb_3_ is galactose α 1-4 galactose β 1-4 glucosyl ceramide. The terminal gal α 1-4 gal is bound by the VT B subunit pentamer, but the lipid moiety is necessary for high affinity binding. While Gb_3_ is the receptor for VTs from human EHEC pathogens, VT2e from the pig EHEC binds Gb_4_, the next member of the globoseries GSLs which contains an additional β 1-3 galNAc (DeGrandis et al., 1989). Interestingly, although VT1 and VT2 do not bind Gb_4_, they bind (non-physiological) deacetylGb_4_ (terminal free amino sugar) in preference to Gb_3_ (Nyholm et al., 1996). Differential chemical substitution of this free amine has a remarkable and varied effect on the binding of different VTs (Mylvaganam et al., 2015). The binding affinity for the lipid-free gal α 1-4 gal disaccharide is many orders less than for Gb_3_ (St. Hilaire et al., 1994). The ceramide lipid component of Gb_3_ (and all GSLs) varies greatly and this has a major effect on VTB:Gb_3_ binding (Pellizzari et al., 1992; Kiarash et al., 1994). The orientation of the carbohydrate of membrane GSLs is dependent on both the lipid composition of the GSL itself and the membrane in which it is embedded (Nyholm et al., 1989; Nyholm and Pascher, 1993). Moreover, GSLs accumulate in (detergent resistant) cholesterol enriched lipid rafts (Hooper, 1999; Legros et al., 2017).

## Retrograde Transport

**Figure 1:**
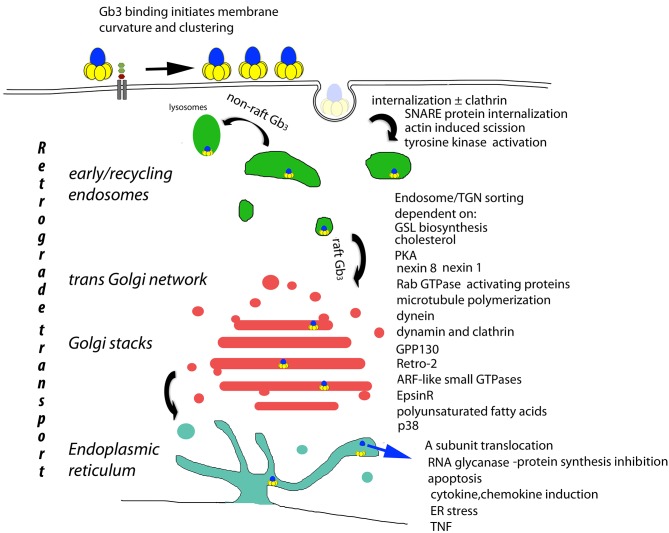
Basis of cellular VT cytopathology. The retrograde transport of plasma membrane bound VT (Römer et al., 2007) is the central determinant of cytopathology. Receptor mediated endocytosis (Sandvig et al., 1989; Khine et al., 2004; Bujny et al., 2007; Rydell et al., 2014; Renard et al., 2015), tyrosine kinase activation (Katagiri et al., 1999; Mori et al., 2000; Lauvrak et al., 2005; Utskarpen et al., 2010), non-raft Gb_3_ bound VT lysosomal transit (Falguieres et al., 2001) raft Gb_3_ bound VT endosomal/TGN transit [and A subunit ER/cytosolic transport] (Lord et al., 2003; Spooner and Lord, 2012) are all essential to end stage cytopathology (Brigotti et al., 1997; Foster et al., 2000; Fujii et al., 2003; Yamasaki et al., 2004; Jandhyala et al., 2008; Stearns-Kurosawa et al., 2010; Leyva-Illades et al., 2012; Debernardi et al., 2018). These control points provide candidates for the prevention of VT cytopathology. However, they also control retrograde transport in general, and although no physiological substrate for retrograde transport has yet been described, the potential for side effects persists.

The presence of Gb_3_ in lipid plasma membrane rafts is required for the retrograde transport of internalized VT to the ER (Falguieres et al., 2001). Non-raft Gb_3_ targets internalized VT to lysosomes for degradation. Receptor mediated VT cell internalization is via clathrin dependent and independent mechanisms (Khine et al., 2004). The multivalent B subunit pentamer binding to cell surface raft and model membrane Gb_3_ has been shown to cluster Gb_3_ (Khine and Lingwood, 1994; Windschiegl et al., 2009; Pezeshkian et al., 2017) which causes subsequent energy and clathrin independent tubular invaginations (negative membrane curvature) (Römer et al., 2007; Bosse et al., 2019) (Figure 1). This effect is Gb_3_ unsaturated fatty acid dependent and does not involve cytoskeletal components (Römer et al., 2007). Pinching off of the vesicles occurs after actin mediated, cholesterol dependent membrane domain reorganization (Römer et al., 2010). Membrane incorporation of lysophospholipids with large head groups blocks VT membrane Gb_3_ binding and can reverse Gb_3_ bound VT (Ailte et al., 2016). These phospholipids induce a positive membrane curvature which may prevent the negative membrane curvature induced by VT-Gb_3_ binding.

Control of endosomal VT/Gb_3_ sorting to the transGolgi network for retrograde transport to the Golgi and ER, is achieved by a large array of trafficking regulators (Figure 1). However, cell bound VT1 and VT2 reach this sorting pathway by different routes (Tam et al., 2008). The A subunit is proteolytically clipped by furin (Lea et al., 1999) in the Golgi (Voorhees et al., 1995), but the C terminal disulfide bond holding the A1 and A2 peptides together is only cleaved in the ER where the A1 subunit separates from the B pentamer, and, via its newly exposed C terminal misfolded protein-mimic sequence (Hazes and Read, 1997), hijacks the ER chaperone quality control system to be unfolded and transported via the ER dislocon into the cytosol (Yu and Haslam, 2005; Tam and Lingwood, 2007) for inhibition of protein synthesis/cell killing via RNA glycanase activity (Endo and Tsurugi, 1987). Since this dislocon is key in ER associated degradation of misfolded proteins (Hebert et al., 2010), this hijacking can be used therapeutically in protein misfolding diseases (Adnan et al., 2016).

## GB_3_ Binding

In the human renal glomerulus, Gb_3_ is in lipid rafts whereas in tubular cells, Gb_3_ is in the non-raft fraction (Khan et al., 2009). This correlates with the glomerular site of primary pathology in eHUS. However, the interaction of cholesterol with GSLs can markedly restrict the orientation of the membrane GSL carbohydrate moiety for ligand binding (Mahfoud et al., 2010; Yahi et al., 2010; Lingwood et al., 2011).

Site specific mutagenesis and molecular modeling from the B subunit crystal structure (Stein et al., 1992) identified 3 potential Gb_3_ binding sites (Nyholm et al., 1989; Ling et al., 1998; Bast et al., 1999; Soltyk et al., 2002). Site 1 is in the interface between B subunits, site 2 is a shallow groove on the membrane apposed B subunit surface and site 3 is the single central tryptophan34. Thermodynamically site 1 is the high affinity site (Nyholm et al., 1996) but cocrystalization with a gal α 1-4 gal carbohydrate showed site 2 was most frequently occupied (Ling et al., 1998). It remains likely that site 1 is the Gb_3_ glycolipid binding site but site 2 has been targeted using gal α 1-4 gal β 1-4 glc sugar derivatives as a potential prophylactic approach (Ling et al., 1998).

## Receptor Analogs

Synsorb p^k^ was the first wherein gal α 1-4 gal β 1-4 glc oligosaccharide was coupled to an inert silica matrix (Armstrong et al., 1991). This was effective to block cytotoxicity in cell culture but not in a randomized clinical trial (Armstrong et al., 1995; Trachtman et al., 2003). Multimeric derivatives (Watanabe et al., 2004; Nishikawa et al., 2005; Jacobson et al., 2014; Matsuoka et al., 2018) [particularly the pentameric “starfish” array (Kitov et al., 2000; Mulvey et al., 2003)] have been used to increase binding avidity generating a “sandwich” whereby the polymeric galactose α 1-4 galactose disaccharide binds to two B subunit pentameric arrays (Kitov et al., 2000). This sandwich structure prevents cell surface Gb_3_ binding to block toxicity. Although effective in cell culture and the mouse to prevent VT cytotoxicity (Kitov et al., 2008a), the compound has yet to be tested clinically as a prophylactic for HUS.

Gb_3_ carbohydrate multimerically coupled to a series of acrylamide polymers also proved effective inhibitors of VT cytopathology in cell culture and in mice (Nishikawa et al., 2005; Watanabe et al., 2006; Watanabe-Takahashi et al., 2010). A Gb_3_-trehalose acrylamide copolymer was also effective against VT in cell culture and the mouse model (Neri et al., 2007a). However acrylamide is toxic *in vivo* (Tareke et al., 2002). In optimization studies of these complexes, it was found the VT 1 and VT2 bind to completely different regions of the Gb_3_ oligosaccharide (Watanabe et al., 2006), a result consistent with our earlier observation that VT1 and VT2 bind preferentially to different conformers of Gb_3_ (Nyholm et al., 1996). A tetravalent peptide was developed (Nishikawa et al., 2006) which prevented the traffic of VT to the ER in cell culture and prevented VT2-induced fluid accumulation in rabbit ileal loops (Watanabe-Takahashi et al., 2010). Gb_3_ oligosaccharides complexed with cyclodextrin proved high affinity receptors for both VT1 and VT2 (Zhang et al., 2017) but not as yet reported for neutralization. Other Gb_3_ mimics have been selected from peptide libraries (Miura et al., 2004, 2006) which are bound by VTs (and antiGb_3_). These were protective in cell culture. However, peptides can be immunogenic and were not tested clinically. Gb_3_ sugar coupled to phosphatidylethanolamine in a liposomal format also proved effective against VT in cell culture (Neri et al., 2007b; Detzner et al., 2019). A unique Gb_3_ sugar based heterobifunctional crosslinker was made to decorate the pentameric serum amyloid P to block VT1/VT2 *in vitro* but rapid clearance prevented *in vivo* efficacy (Kitov et al., 2008b). A similar construct using a GalNAc instead of a Gal Gb3 trisaccharide selectively blocked VT2 *in vivo* (Jacobson et al., 2014).

The sugar sequence of Gb_3_ is mimicked by the carbohydrate moiety of some bacterial lipooligosaccharides (Mandrell and Apicella, 1993). The bacterial β-galactosyl and α-galactosyl transferases responsible from *Neiseria* were cloned into a commensal *E.coli* expressing glucose terminating LPS (Paton et al., 2000). This *E.coli* proved effective to protect cells and animal models against VT pathology (Paton A. W. et al., 2001). A similar Gb_4_-LPS *E.coli* was protective in pig edema disease (Paton A. W. et al., 2001; Hostetter et al., 2014). These constructs were further modified to be suitable for clinical trial (Paton J. C. et al., 2001) and non-genetically modified “Gb_3_” expressing bacterial “ghosts” also were effective (Paton et al., 2015), but as yet no clinical trials have been reported.

Our approach was to target the B subunit Gb_3_ glycolipid binding site, site1 (Nyholm et al., 1996). Since the binding affinity for the lipid-free *trisaccharide* of Gb_3_ is so low, we attempted to generate a “water soluble” Gb_3_ analog (Mylvaganam and Lingwood, 1999). We found that substituting the fatty acid of GSLs with an adamantane frame generated species which partitioned into water yet retained similar hydrophobicity as monitored by thin layer chromatogram mobility (Lingwood and Mylvaganam, 2003). Although the fatty acid is important in VT-Gb_3_ binding (Kiarash et al., 1994; Pezeshkian et al., 2015), these species (unlike the free sugar) retained the biological activity of the membrane embedded GSL in solution (Lingwood et al., 2006). AdaGlcCer and adaGalCer proved effective inhibitors of cellular GSL biosynthesis (Kamani et al., 2011), while adaSGC was an effective mimic of SGC (3-sulfogalactosyl ceramide), binding to Hsp70 (Mamelak and Lingwood, 2001), thereby inhibiting its ATPase activity (Whetstone and Lingwood, 2003) and chaperone action in cells (Park et al., 2009). Hsp70-SGC binding promotes aggregation of Hsp70 for high affinity peptide binding and blocks ATP-mediated peptide release (Harada et al., 2015).

AdamantylGb_3_ is a highly effective receptor for the VTB subunit (Mylvaganam and Lingwood, 1999; Lingwood et al., 2006). AdaGb_3_ is bound by VT2 more effectively than Gb_3_ (Saito et al., 2012). AdaGb_3_ blocks VT –Gb_3_ and cell binding *in vitro* (Mylvaganam and Lingwood, 1999). We made several lipid derivatives of adaGb_3._ However, adaGb_3_ and derivatives can incorporate into Gb_3_ negative cell lines and induce VT1/2 sensitivity (Saito et al., 2012). This is interesting, since the different analogs can differentially subvert the intracellular traffic of VT (Saito et al., 2012), but is not a feature consistent with a therapeutic. We therefore made an adamantyl bis Gb_3_ which has two deacylGb_3_s linked to a single central adamantane frame. This protected cells from VT more effectively than adaGb_3_ and was not incorporated into receptor negative cells (Saito et al., 2012). Despite protection against VT in cell culture, subsequent *in vivo* mouse susceptibility studies with adabisGb_3_ showed that treated mice showed a more rapid VT-induced pathology than control mice. A similar effect has been reported for lysoGb_3_ containing liposomes *in vivo* (Takenaga et al., 2000). While the basis of this effect in not clear, it may relate to the potential of multivalent VT to show cooperative receptor binding (Peter and Lingwood, 2000).

## VT and eHUS

The pathology of eHUS is complex. In the baboon model, sterile VT was clearly shown to induce HUS following intravenous administration (Siegler et al., 2003). The question of how VT enters the blood stream following gastrointestinal EHEC infection, is still unclear. VT is difficult to detect in patient blood after EHEC infection but can be detected in the acute phase (He et al., 2015; Yamada et al., 2019). However, these blood concentrations are far lower than needed to cause HUS in the baboon. Moreover, if the VT dose inducing HUS in baboon was divided into 4 (still much higher than ever detected in eHUS patients) and given every 12 h, no HUS pathology was observed (Siegler R. et al., 2001).

Significantly, Gb_3_ is upregulated in many human cancers (LaCasse et al., 1999; Kovbasnjuk et al., 2005; Distler et al., 2009; Stimmer et al., 2014). In light of the baboon data indicating there could be a “safe” dose for sterile VT, we (Farkas-Himsley et al., 1995; Arab et al., 1999; Arbus et al., 2000) and later others (Gariepy, 2001; Falguieres et al., 2008; Devenica et al., 2011; Engedal et al., 2011), have proposed VT could be used as the basis of an antineoplastic.

## Other Factors in eHUS

It is clear that other systemic factors are at play in eHUS. One of these is bacterial LPS, which augments baboon VT renal toxicity (Siegler R. L., et al., 2001) and renal Gb_3_ (Clayton et al., 2005). LPS can induce proinflammatory cytokines TNF and IL1 β (Eggesbo et al., 1996) which in turn, can increase cell Gb_3_ synthesis (Warnier et al., 2006) and VT sensitivity (Louise and Obrig, 1991). VT itself can induce TNF production (van Setten et al., 1996) by monocytes. These cells are not sensitive to VT toxicity but the A subunit is required (Foster et al., 2000) (Figure 1). This cytokine has been recently shown to play a role to set VT sensitivity in eHUS (Brigotti et al., 2018; Lafalla Manzano et al., 2019).

VT interaction with neutrophils has also been suggested to be important. Gb_3_ is not expressed in the human colonic epithelium (Miyamoto et al., 2006) and so the mechanism by which gastrointestinal VT gains systemic access remains to be answered. Several studies suggest a VT-neutrophil transport function (Hurley et al., 2001; Griener et al., 2007; Brigotti et al., 2008, 2010). Other work, however, concluded VT does not bind human neutrophils (Flagler et al., 2007; Geelen et al., 2007), although mouse neutrophils were bound (Fernandez et al., 2000, 2006) and can target mouse kidney (Roche et al., 2007). Nevertheless, recent studies show neutrophil released traps [NETs -anti infectious/inflammatory response (Boeltz et al., 2019)] are increased (Ramos et al., 2016) and their degradation reduced (Leffler et al., 2017) in eHUS.

Since complement insufficiency plays a central role in idiopathic thrombocytopenia and atypical HUS (Nielsen et al., 1994), VT activation of the alternative complement pathway (Morigi et al., 2011) has become considered of potential importance in eHUS (Karpman and Tati, 2016; Fakhouri and Loirat, 2018). However, these interactions of VT with different components of the blood system should be reviewed in light of the differences showed for the binding of native vs. proteolytically clipped VT recently reported (Brigotti et al., 2019).

## New Therapeutics

### Complement

The treatment of acquired or congenital thrombocytopenia with Eculizumab, an anti-C5 monoclonal antibody which blocks complement activation, has proven successful (Pecoraro et al., 2015; Bitzan et al., 2018). Since VT can activate complement, this approach has been tested for efficacy in eHUS (Rasa et al., 2017; Walsh and Johnson, 2019). Therapeutic effect has been shown, particularly for patients with neurological symptoms (Percheron et al., 2018) but results overall are, as yet, highly variable (Loos et al., 2018; Monet-Didailler et al., 2019).

### Stem Cells

Advantage has recently been taken of the huge effort to develop stem cells as disease therapeutics. Muse (multilineage differentiating stress enduring) cells are a small non-tumorigenic, non-immunogenic component of the mesenchymal stem cell fraction (Kuroda et al., 2010) which can be isolated from many sources (Leng et al., 2019), including bone marrow, adipose tissue and fibroblasts. Muse cells home to sites of tissue damage and there differentiate to regenerate the damaged tissue type (Young, 2018). This includes neuronal cells (Nitobe et al., 2019). There are many current clinical trials based on these cells (Dezawa, 2018; Kuroda et al., 2018).

Human Muse cells were used to rescue a NOD-SCID mouse model of EHEC (VT2) induced neurological disease (Ozuru et al., 2019). Forty eight hour after gastrointestinal EHEC treatment Muse cell i.v. injection resulted in complete survival and lack of weight loss, whereas >50% of untreated mice died of encephalopathy. Splitting the Muse cell dosage and treating at 24 and 48 h post-infection did not give protection. Human Muse cells were found in the mouse brain and the level of VT2 activated astrocytes significantly reduced. Knockdown of G-CSF largely ablated the protection.

These studies bode well for the development of an effective means to prevent/treat VT-based disease in the future.

## Author Contributions

The author confirms being the sole contributor of this work and has approved it for publication.

### Conflict of Interest

CL is a founder of ERAD Therapeutic which is developing bacterial toxoids as treatment for protein misfolding diseases.
